# Composite diffuse large B-cell lymphoma and peripheral T-cell lymphoma: a case report with two-year follow-up and literature review

**DOI:** 10.3389/fonc.2024.1272209

**Published:** 2024-03-11

**Authors:** Jiwei Gu, Juan Qian, Xin Cao

**Affiliations:** Department of Hematology, Affiliated Hospital of Nantong University, Nantong, China

**Keywords:** diffuse large B cell lymphoma, peripheral T-cell lymphoma, treatment, composite lymphoma, intestine

## Abstract

Composite lymphoma is an uncommon type of lymphoid malignancy, and those consisting of concurrent diffuse large B-cell lymphoma (DLBCL) and peripheral T-cell lymphoma, not otherwise specified (PTCL-NOS) in the same organ are rare. Here, we report a case of a 75-year-old male patient admitted to our emergency department with intestinal obstruction presenting with abdominal pain and vomiting. He underwent partial resection of the small intestine under general anesthesia, and subsequent histopathology confirmed the mass to be composite DLBCL and PTCL-NOS. The patient received chemotherapy with a rituximab-based regimen and achieved complete remission (CR). However, the recurrent disease presented with obstruction again ten months after treatment. He refused a second surgery, but salvage treatment was not effective. The patient survived for 20 months after diagnosis. In addition, we did a literature review to understand the clinical features, pathology, treatment, and prognosis of this type of composite lymphoma.

## Introduction

Composite lymphoma (CL) is an uncommon type of lymphoid malignancy, accounting for approximately 1.0%–4.7% of all lymphomas (1). Those consisting of concurrent B- and T-cell tumors are especially rare. Diffuse large B-cell lymphoma (DLBCL) is the most common and heterogeneous B-cell neoplasm, generally expressing CD20. Peripheral T-cell lymphoma (PTCL) is a group of highly heterogeneous invasive non-Hodgkin’s lymphoma (NHL) originating from mature T cells or T cells in the thymus. T cells generally do not express CD20. However, a small subpopulation of T cells also was found expressing CD20. They may be found in healthy controls, autoimmune diseases, and hematological malignancies (2). CD20 expression in PTCL, not otherwise specified (PTCL-NOS), has rarely been reported in the literature, and its clinical significance has not been established yet (3, 4). Twelve cases of DLBCL and PTCL-NOS that occur simultaneously in the same tissue have been reported (4–13). Here, we describe a composite DLBCL and PTCL-NOS case with CD20 expression who presented to the hematology department with intestinal obstruction.

## Case presentation

**Figure 1:**
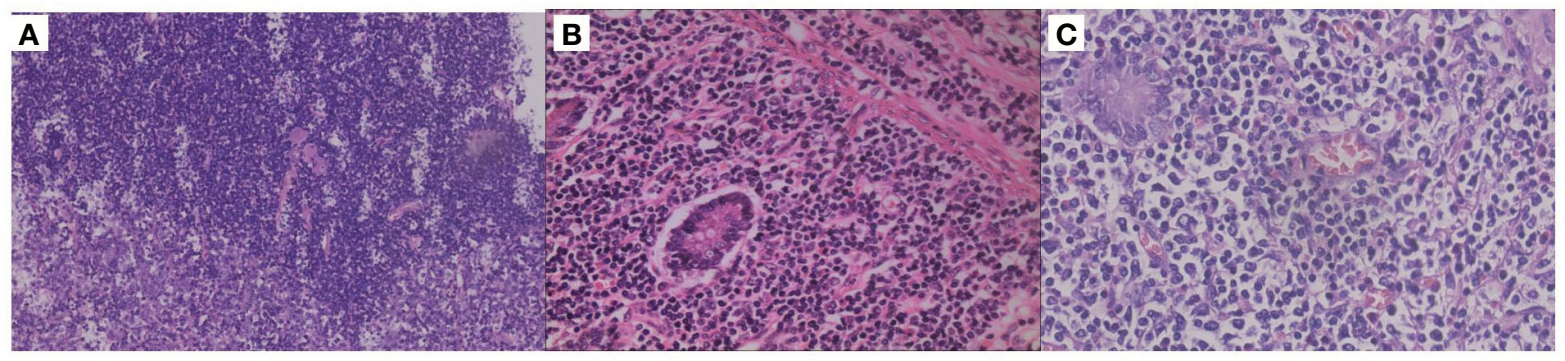
Histopathological results of different parts. **(A)** Hematoxylin-eosin (HE) staining (100X) of the junction between the diffuse large B-cell lymphoma (DLBCL) and peripheral T-cell lymphoma, not otherwise specified (PTCL-NOS) in the intestinal mucosa. **(B)** HE staining of intestinal mucosa with PTCL-NOS (×400). **(C)** HE staining of the intestinal mucosa with DLBCL (×400).

**Figure 2:**
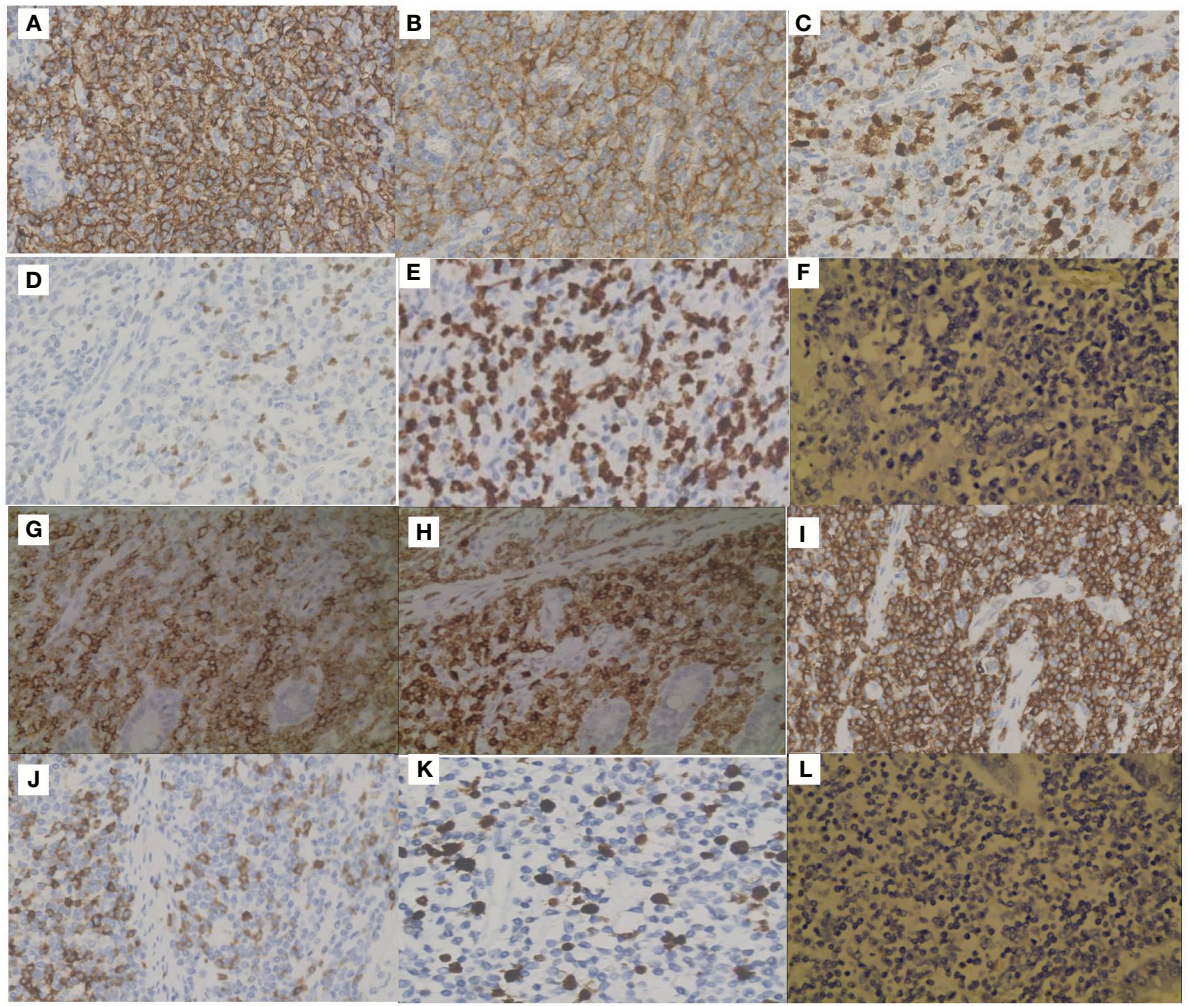
Immunohistochemical staining (IHC) of the two components. Positive IHC staining of CD20 **(A)**, CD10 **(B)**, MUM1 **(C)**, Bcl-6 **(D)**, Ki67 **(E)** and negative staining of EBER **(F)** in DLBCL. Positive IHC staining of CD20 **(G)**, CD3 **(H)**, CD5 **(I)**, CD7 **(J)**, Ki67 **(K)** and negative staining of EBER **(L)** in PTCL-NOS.

**Figure 3:**
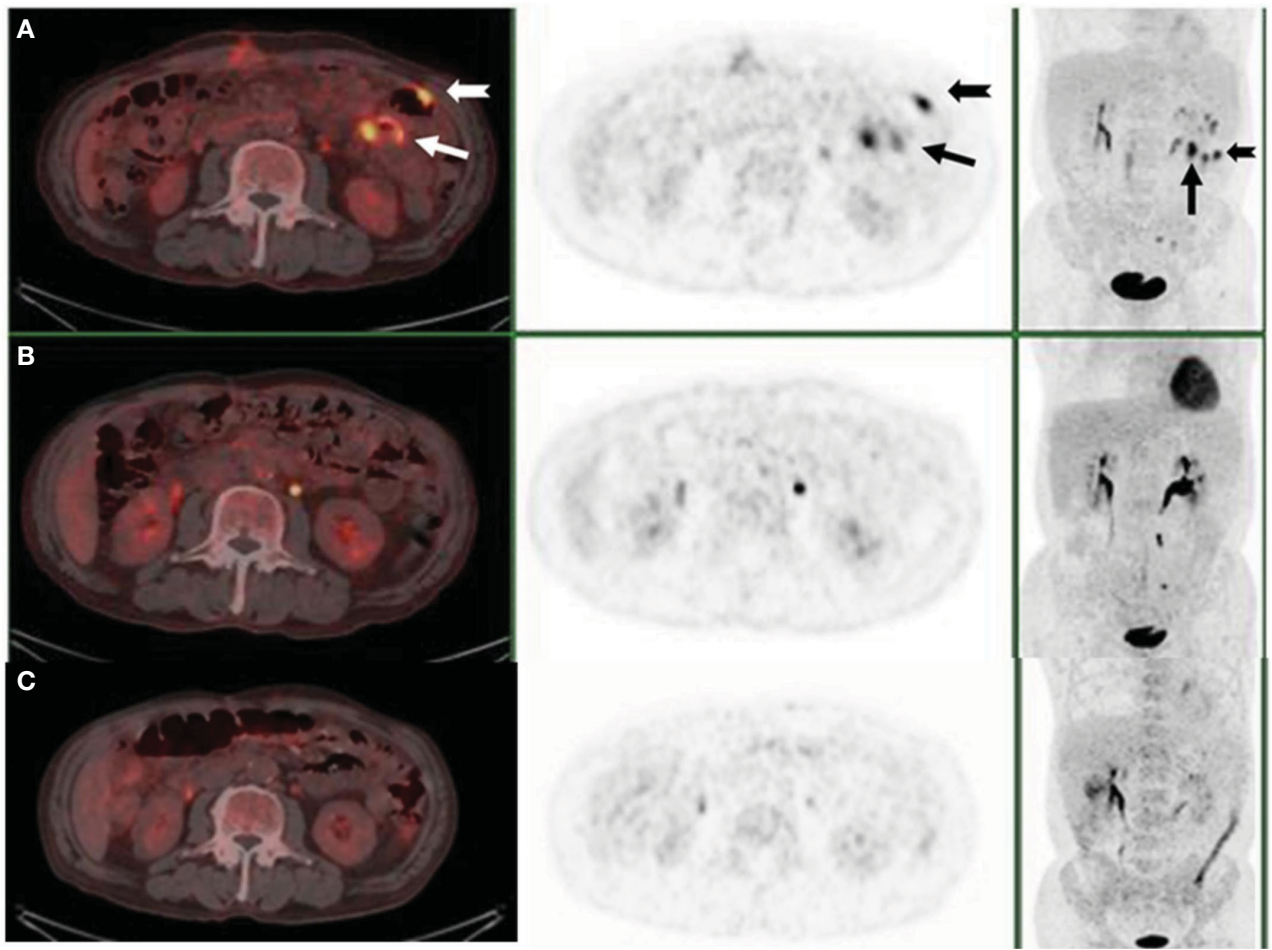
PET/CT scan after small bowel resection **(A)**, three cycles of R-CHOP **(B)**, and five months after treatment **(C)**. The arrows demonstrated the lesions where abdominal masses disappeared.

In October 2019, a 75-year-old man was admitted to the emergency department with worsened abdominal pain accompanied by vomiting. He had abdominal discomfort, night sweats, and loss of appetite and weight for two weeks. A computed tomography (CT) scan showed the thickened upper jejunal wall accompanied by obstructive dilatation of the proximal intestine and multiple enlarged lymph nodes. He received partial small intestine resection and was transferred to the hematology department due to the intraoperative pathology indicating malignant lymphoma. Physical examination didn’t show palpable lymph nodes. He had a ten-year history of hypertension, hyperglycemia, and psoriasis with a penicillin allergy. Complete blood cell count showed mild lymphocytopenia: white blood count (WBC): 2.8×10^9^/L, hemoglobulin concentration (Hb): 109g/L. A stool routine test was weakly positive for occult blood. Other results included lactate dehydrogenase (LDH) 189U/L (0-247U/L), β2-microglobulin (β2-MG) 3.00 ug/ml (1.00-3.00 ug/ml). Epstein-Barr virus (EBV) test showed EBV early antigen IgM (-), EBV viral capsid antigen (VCA) IgM (-), EBV-VCA IgG (+), EBV core antigen IgG (+), EBV-DNA (-). No apparent abnormalities were found on bone marrow biopsy, smear, or flow cytometry. Histopathology of small bowel resection (**Figures 1**, **2**) is as follows. (1) DLBCL was found in the small intestine (1 cm, 6.5 cm, and 16 cm away from the incision), which was germinal center B-cell-like (GCB) DLBCL according to the Hans algorithm. Immunohistochemical (IHC) studies of the tumors showed that the lesion was positive for CD20, CD79a, CD21, Mum-1, Bcl-6, BCL2, CD10, and negative for CD3, CD5, CD43, and CyclinD1. The Ki67 proliferation index was 60%. EBV encodes *in situ* hybridization of small RNA (EBER) was negative. (2) The thickened area of small intestine mucosa indicated PTCL-NOS next to the DLBCL. IHC showed tumor cells were positive for CD2, CD3, CD4, CD5, CD7, CD20, PAX5, and negative for CD56, TdT, EBER with Ki67 proliferation index of 30%. (3) Three of the 30 mesenteric lymph nodes were infiltrated with PTCL-NOS. IHC was positive for CD2, CD3, CD5, CD43, CD20, Bcl2, while negative for CD10, CD79a, Mum1, Bcl6, CyclinD1, PAX5, OCT2, MPO, CD34, TdT. CD7 was lost in part of the tumor cells. The Ki67 proliferation index is about 10%. The immunoglobulin heavy chain gene (IgH) rearrangement test in DLBCL was positive, and IgH rearrangement and T cell receptor (TCR) rearrangement in the part of PTCL-NOS were negative. We arranged an ^18^F- fluorodeoxyglucose (FDG) positron emission computed tomography (PET-CT) for him. The images showed that the operative area of the small intestine was slightly disorganized. A slight thickening of the intestinal wall at the anastomosis and its adjacent area was accompanied by a significant progressive increase of FDG uptake, suggesting the infiltration of residual lymphoma lesions. The increased FDG uptake of the multiple segments of the small intestine in the left abdomen and lymph nodes in the pelvic mesenteric indicated the involvement of lymphoma (**Figure 3**). Combined with clinical manifestations and laboratory findings, he was diagnosed with composite DLBCL and PTCL-NOS, and the Eastern American Cancer Collaboration (ECOG) physical condition score was 2. He received three cycles of R-CHOP (Rituximab, Cyclophosphamide, Epirubicin, Vindesine, Prednisone) and achieved partial remission (PR) (**Figure 3B**). We tried to add Chidamide but failed with severe gastrointestinal reaction and fatigue. The patient took rituximab monotherapy in the fourth course due to fever and neutropenia. Then, he continued three cycles of R-CHOP chemotherapy; the last chemo date was in March 2020. On August 20, 2020, a PET-CT scan showed the disease was in metabolic remission with a Duveil score of 3 (**Figure 3C**). There were no complaints of discomfort in the clinic and no complaints of discomfort during follow-up until March 2020. However, he presented with obstruction again at the end of January 2021, about ten months after treatment, and a CT scan confirmed recurrent disease in the small intestine. He refused a second surgery or endoscopy. Chemotherapy with R-CHOPE (etoposide) relieved his bowel obstruction, however, with increased pleura effusion. Salvage treatment R-GDP (Rituximab, gemcitabine, cis-platinum, Prednisone) was also ineffective. The patient died in May 2021 at a local hospital. He survived for 18 months after diagnosis.

## Discussion

Custer first introduced the term CL in 1954. Kim et al. further modified the concept of CL to the simultaneous occurrence of more than one histologically distinct lymphoma in the same anatomical organ in 1977 (14). Most CLs reported in the literature are classical Hodgkin’s lymphoma (HL) combined with non-Hodgkin’s lymphoma (NHL) or two different B-cell NHL, while the concurrence of B-NHL and T-cell lymphoma is rare (6, 15). DLBCL is the most common B-cell member, followed by hairy cell leukemia, chronic lymphocytic leukemia/small lymphocytic lymphoma, and splenic marginal zone lymphoma. The most common T-cell components are large granular lymphocytic leukemia and angioimmunoblastic T-cell lymphoma (4, 16–18). However, the co-occurrence of DLBCL and PTCL-NOS is rare, with 12 cases reported in the literature (6–13). In this case, it occurred in the small intestine with abnormal expression CD20 in PTCL-NOS.

In addition to our case, twelve cases of CL with DLBCL and PTCL-NOS have been reported. We summarized the clinicopathological features of these cases in **Table 1**. The male-to-female ratio was 2.25:1, ranging from 25 to 91 years (median: 67). Three were Asian, and ten were Caucasian. The occurrence site included the larynx (1 case), lymph node (3 cases), small intestine (2 cases), and bone (1 case), while the data of the remaining 6 cases were missing. Four of the ten patients had a history of hematological diseases, including polycythemia vera, Hodgkin’s lymphoma, cutaneous T-cell lymphoma, and indolent B lymphoma. Of the three reported cases that provided the CD20 information, two were positive, and one was negative. Three patients were positive for EBER in DLBCL while negative for PTCL-NOS components. Six patients were negative for EBER in both DLBCL and PTCL-NOS. In another case, EBER was weakly positive in DLBCL but not in PTCL-NOS. The other three cases didn’t mention the EBER result. Six of ten patients showed positive TCR gene rearrangement. Eight out of ten patients showed IgH gene rearrangement. Five patients received chemotherapy, one with chemotherapy combined with radiotherapy, one used topical therapy, one refused treatment, and five cases did not show the details. One patient achieved PR after four cycles of chemotherapy but died of surgery. One patient died of cachexia six months after topical treatment. Six patients were unknown about the prognosis. The median follow-up time for the seven cases was eleven months (1-101 months).

**Table 1:** Clinical and pathological features of 13 patients with composite diffuse large B-cell lymphoma (DLBCL) and peripheral T-cell lymphoma, not otherwise specified (PTCL.NOS).

Year	Country	Sex	Age	Site	Other sites involved	Past medical history	CD20 expression of PTCL.NOS	EBER		Molecular information	Treatment and follow-up	Citation
								DLBCL	PTCL.NOS			
2002	Germany	M	25	N/A	Lymph nodes, spleen, epidural space	N/A	N/A	+	-	TCR+, IgH+	Polychemotherapy, alive after 101 months	(8)
2002	Germany	F	89	N/A	Lymph nodes	N/A	N/A	+	–	TCR-, IgH-	Untreated, lost to follow-up	(8)
2002	Germany	F	91	N/A	Lymph nodes, bone marrow, skin	N/A	N/A	+	-	TCR+, IgH+	Intrathecal injection with methotrexate, follow-up for six months, died of cachexia.	(8)
2005	America	M	35	Tibia	Tibial soft tissue	None	N/A	–	–	TCR-,IgH+	N/A	(6)
2006	America	M	49	Ileum	None	Gastroesophageal reflux	-	-	-	TCR+, IgH+	Achieve CR after six cycles of R-CHOP, with no disease progression for 15 months.	(9)
2008	Italy	M	67	Lymph node	Bone marrow	Tuberculosis	N/A	–	–	TCR-, IgH+	Achieved CR after six cycles of R-CHOP and radiation, with no disease progression for 6 months.	(10)
2012	America	M	43	laryngeal	Lung, stomach, and mesenteric lymph nodes	Hodgkin lymphoma	N/A	+(weak)	N/A	TCR+, IgH+	Achieved PR after 4 cycles of R-CHOP but died of bleeding at the surgical site.	(12)
2011	Japan	F	67	cervical lymph node	Bone, epidural space	None	Positive	–	–	TCR+, IgH-	Achieve CR after six cycles of R-CHOP and radiation, with no disease progression for 8 months.	(7)
2016	America	M	82	N/A	N/A	**polycythemia vera**	N/A	+ (Unspecified)		N/A	N/A	(13)
2016	America	F	33	N/A	N/A	Cutaneous T lymphoma	N/A	-(Unspecified)		N/A	N/A	(13)
2016	America	M	70	N/A	N/A	**un-specific**	N/A	+ (Unspecified)		N/A	N/A	(12)
2019	Japan	M	73	Axillary lymph nodes	N/A	Indolent B lymphoma	N/A	–	–	TCR+, IgH+	N/A	(11)
2019	China	M	75	Small intestine	Mesenteric lymph nodes	Psoriasis	Positive	-	-	TCR-, IgH+	Achieved CR after six cycles of R-CHOP, with disease progression after 11 months, and died four months later.	This case

N/A, Not Applicable; EBER, Epstein-Barr virus encodes in situ hybridization of small RNA; TCR, T cell receptor rearrangement; IgH, Immunoglobulin gene rearrangement; R-CHOP, Rituximab, cyclophosphamide, doxorubicin, vincristine, prednisone; +, positive; -, negative.

The pathogenesis of CL is still unclear. Scholars proposed some hypotheses for the coexistence of T-cell and B-cell tumors in the same tissue (4, 5, 8, 19, 20). One of the most commonly mentioned is the virological hypothesis. EBV infection may cause simultaneous or sequential transformation of B cell and T cell components, leading to the development of the two types of lymphoma (8, 20). On the one hand, the expression of EBV antigen in neoplastic B cells may stimulate the proliferation of T cells and eventually transform into T-cell lymphoma via clonal selection. Alternatively, the first appearance of T-cell lymphoma may also result in a deficiency in innate immunity that renders host B cells more susceptible to EBV infection, leading to transformation into B-cell neoplasms. In such cases, the patient’s T-cell and B-cell tumor components tend to be EBER positive (8, 21). Although this patient was positive for EBV-associated IgG, the pathology indicated that EBER was negative for both T and B cell components. Virological theories still can not explain our case and other instances of CL without a virological basis.

Another proposed mechanism in this era of high-throughput genome sequencing is the hypothesis of acquired oncogene mutations in lymphoid progenitor cells. Wang et al. (5) reported a case of CL composed of PTCL and mantle cell lymphoma, and both components were positive for the CCND1/IgH fusion gene and cyclin D1 overexpression. Therefore, the authors believed there were specific genetic variations in lymphoid progenitor cells. Then, other genomic modifications evolve into heterogeneous subclones, resulting in the co-development of T - and B-cell tumors. Given the clonal correlation between the two tumor components, authors assumed that the two tumors may share a co-progenitor cell or grow in the same microenvironment. The progress of genomics provides an ideal tool for studying the clonal origin and clonal evolution of similar composite lymphoid tumors (22). It is helpful to investigate the tumor lineage of CL by analyzing the genomic profiles of B-cell and T-cell tumors. However, the results of high-throughput sequencing of genes for the components of the two tumor cells were lacking in this patient. In conclusion, the pathogenesis of CL of B cell and T cell origin is complex, and there may be multiple pathophysiological pathways (4, 5, 20).

Another feature of our case is the aberrant expression of CD20 in PTCL-NOS. The incidence of CD20 expression in T cell lymphoma reported in the literature was about 5-8% (23). There are several hypotheses about its pathogenesis. First, as there are a small number of CD20 weakly expressed normal T cells in peripheral blood, bone marrow, and lymph nodes during normal hematopoiesis (24), it is speculated that CD20-positive T cell lymphoma originates from this group of malignant T cell subsets. Second, CD20 expression can be induced by T lymphocytes in the process of stimulation or proliferation and activation *in vitro* (25). Thus, CD20 expression may also be related to the activation of T-cell lymphoma cells. Third, there are progenitor cells with the potential to differentiate into B cells, T cells, and NK cells in cord blood (26), so CD20-positive PTCL may also be the product of the malignant transformation of progenitor cells at a stage of differentiation. In our case and another case reported in the literature (7), the intensity of CD20 in PTCL-NOS was weaker than that of DLBCL counterparts. Therefore, we suspect these two components may have different cellular origins, but the exact mechanism needs further investigation.

Chemotherapy therapy and radiation are common and effective treatments for lymphoma. In CL, the choice of the treatment regimen and the patient’s prognosis is mainly based on the more aggressive type of lymphoma (27). In this case, DLBCL exists simultaneously with PTCL-NOS. Therefore, this patient was treated with rituximab and CHOP. Rituximab has been widely used in treating CD20-positive B-cell lymphoma, while the efficacy in CD20-positive T-cell lymphoma is still unclear. Shao et al. reported a T-cell lymphoma with CD20 expression showing excellent response to rituximab with gemcitabine, oxaliplatin, and L-asparaginase (R-pGEMOX) instead of initial chemotherapy without rituximab (28). Mangogna A. et al. provide a PTCL-NOS case with aberrant expression with CD20 and CD79a who did not benefit from rituximab-based chemotherapy (29). Kakinoki et al. considered that the effectiveness of rituximab may be associated with the intensity of CD20 expression in T cells, and patients with abundant CD20 expression will benefit the most from treatment with R-based chemotherapy (30). This patient and another case reported in the literature with CD20-positive PTCL-NOS were treated with a standard R-CHOP regimen for six courses and achieved CR. From the literature and our case data, five patients received R-CHOP therapy, four patients achieved CR, one patient achieved PR, and the median follow-up time was 11 months. However, with extended follow-up, our patient relapsed. Unfortunately, he refused to undergo another biopsy, so the type of recurrent lymphoma remains unknown. and he eventually died from the disease.

## Conclusion

In conclusion, the simultaneous occurrence of DLBCL and CD20-positive PTCL-NOS in the same tissue is infrequent in clinical practice, and it is not easy to diagnose and easy to miss and misdiagnose. To correctly diagnose this rare disease, clinicians must work with pathologists carefully, combining multiple detection methods, using as many tissues as possible in the biopsy, and avoiding lymph node puncture. Due to the poor prognosis associated with the simultaneous development of numerous histological types of lymphoma and the lack of data on treatment and outcome, the exact prognosis, treatment options, molecular genetic changes, and the mechanism of the disease occurrence still need to be further studied and explored.

## Data availability statement

The original contributions presented in the study are included in the article/supplementary material. Further inquiries can be directed to the corresponding author.

## Ethics statement

Written informed consent was obtained from the participant/patient(s) for the publication of this case report.

## Author contributions

XC: Data curation, Funding acquisition, Writing – review & editing. JG: Formal Analysis, Writing – original draft. JQ: Investigation, Resources, Writing – review & editing.
